# Dust emission reduction enhanced gas-to-particle conversion of ammonia in the North China Plain

**DOI:** 10.1038/s41467-022-34733-4

**Published:** 2022-11-12

**Authors:** Yongchun Liu, Junlei Zhan, Feixue Zheng, Boying Song, Yusheng Zhang, Wei Ma, Chenjie Hua, Jiali Xie, Xiaolei Bao, Chao Yan, Federico Bianchi, Tuukka Petäjä, Aijun Ding, Yu Song, Hong He, Markku Kulmala

**Affiliations:** 1 Aerosol and Haze Laboratory, Advanced Innovation Center for Soft Matter Science and Engineering, Beijing University of Chemical Technology, 100029 Beijing, China; 2 Hebei Technological Innovation Center for Volatile Organic Compounds Detection and Treatment in Chemical Industry, Hebei Chemical & Pharmaceutical College, Shijiazhuang, 050026 China; 3 Hebei Provincial Academy of Environmental Sciences, Shijiazhuang, 050037 China; 4 Institute for Atmospheric and Earth System Research, Faculty of Science, University of Helsinki, Helsinki, 00014 Finland; 5 Joint International Research Laboratory of Atmospheric and Earth System Sciences, School of Atmospheric Sciences, Nanjing University, Nanjing, 210023 China; 6 State Key Joint Laboratory of Environmental Simulation and Pollution Control, College of Environmental Sciences and Engineering, Peking University, 100871 Beijing, China; 7 Research Center for Eco-Environmental Sciences, Chinese Academy of Sciences, 100085 Beijing, China

**Keywords:** Atmospheric chemistry, Geochemistry

## Abstract

Ammonium salt is an important component of particulate matter with aerodynamic diameter less than 2.5 µm (PM_2.5_) and has significant impacts on air quality, climate, and natural ecosystems. However, a fundamental understanding of the conversion kinetics from ammonia to ammonium in unique environments of high aerosol loading is lacking. Here, we report the uptake coefficient of ammonia (γ_NH3_) on ambient PM_2.5_ varying from 2.2 × 10^−4^ to 6.0 × 10^−4^ in the North China Plain. It is significantly lower than those on the model particles under simple conditions reported in the literature. The probability-weighted γ_NH3_ increases obviously, which is well explained by the annual decrease in aerosol pH due to the significant decline in alkali and alkali earth metal contents from the emission source of dust. Our results elaborate on the complex interactions between primary emissions and the secondary formation of aerosols and the important role of dust in atmospheric chemistry.

## Abstract (web summary)

Liu et al. found that the formation rate of particulate ammonium is slower in the atmosphere than that observed in the laboratory, while it is sped up due to an increase in aerosol acidity driven by an emission reduction of dust in North China Plain.

## Introduction

Ammonia is the most abundant alkaline gas in the atmosphere, with global emissions estimated to be greater than 58 Tg(N) Yr^−1^ ^1^. Both natural and anthropogenic sources, including soils, oceans, fertilizers, livestock, automobiles, biomass burning, etc., contribute to atmospheric NH_3_^2–5^. In the atmosphere, NH_3_ is responsible for neutralizing sulfuric and nitric acids formed through the oxidation of SO_2_ and NO_x_, leading to the formation of NH_4_^+^^6^, which is an important component of atmospheric particulate matter (PM)^7^ and has significant impacts on air quality, climate, and natural ecosystems^8,9^.

Atmospheric concentrations of NH_3_ vary from several to several tens of ppbv (parts per billion in volume)^10^ and show upwards trends over several of the world’s major agricultural regions^11–14^. NH_3_ emissions in China were estimated to be ~10 Tg in the 2000s^9,15,16^ and exceeded the sum of those in the European Union and the United States^9^. Modeling studies revealed that NH_3_ is an essential controlling factor regarding sulfate (SO_4_^2−^)-nitrate (NO_3_^–^)-ammonium (SNA) aerosol and fine particle pollution over China^9,17^. The relative contribution of ammonium nitrate to PM_2.5_ (with an aerodynamic diameter of PM less than 2.5 μm) increases as a function of PM mass concentrations at urban sites^18,19^. The concentrations of NO_3_^–^ and NH_4_^+^ in Beijing increased at rates of 0.8 and 0.6 μg m^−3^ Yr^−1^, respectively, as the concentration of SO_4_^2−^ decreased obviously after 2013^17,20^. Furthermore, ammonia is important for atmospheric new particle formation (NPF) in megacities^21^. Therefore, it is very important to understand the conversion kinetics from NH_3_ to NH_4_^+^ in the atmosphere.

Thermodynamic models assume that NH_3_ uptake and particle neutralization occur within a model time step (usually from several seconds to minutes), as inferred from laboratory studies^22,23^. The NH_3_ uptake kinetics will thus significantly affect the ability of models to properly predict the transport of NH_3_ and NH_4_^+^^23–26^. The uptake kinetics of NH_3_ on aerosol particles are scarce, although a few studies have reported the uptake coefficient (γ_NH3_) or accommodation coefficient (α) of NH_3_ on model particles such as ice^27^, sulfuric acid/H_2_O^22,28–32^, organic acids^28,33^ and organic aerosols (OA)^34^, with the γ_NH3_ values varying from 10^−4^ to 1.0. In the atmosphere, numerous air pollutants have a complicated influence on the uptake kinetics of trace gases, including NH_3_. Liggio et al.^23^ reported that the γ_NH3_ on sulfuric acid aerosols varied from 4 × 10^−3^ to 2 × 10^−4^ when the OA-to-sulfate mass ratio was from 0.14 to 0.55, in contrast to ~1.0 for the organic-free system. Similar retardancy effects were also observed for the uptake of NH_3_ by sulfuric acid aerosols precoated with *n*-hexadecanol^35^ and *n*-hexadecane^36^. The composition and mixing state of atmospheric PM should be more sophisticated than those simulated in the laboratory. However, the uptake coefficient of NH_3_ on real ambient PM is currently lacking. It is also unknown how the conversion kinetics changes in the atmosphere.

In this article, we demonstrate the γ_NH3_ on ambient PM_2.5_ in the North China Plain (NCP) based on long-term field observations. We show that the γ_NH3_ on ambient PM_2.5_ is significantly lower than those on model aerosol particles, while it increases annually in the statistical sense driven by an increase in aerosol acidity. By comprehensively analyzing the chemical composition and source apportionment of aerosol particles, we find that the reduction of alkali and alkali earth metals from dust emissions resulted in an increase in aerosol acidity, although the ratio of sulfate-to-nitrate in PM_2.5_ slightly decreases, followed by the conversion kinetics from NH_3_ to NH_4_^+^. The overall goal of this work is to provide a fundamental understanding of particle-phase NH_4_^+^ formation in the context of decreasing the mass loading of sulfate in aerosol particles.

## Results

### Variations in NH_4_^+^ and NH_3_

**Fig. 1:**
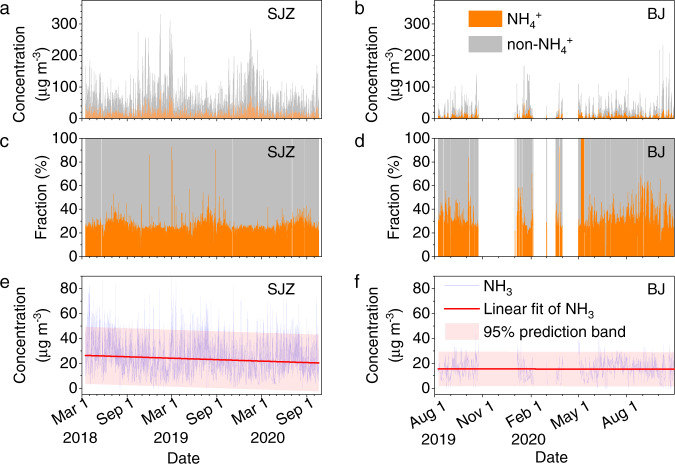
Concentrations of particulate NH_4_^+^, non-NH_4_^+^ ions and gas-phase NH_3_. The time series of (**a**, **b**) mass concentrations of particulate NH_4_^+^ and non-NH_4_^+^ in water-soluble ions, (**c**, **d**) the relative fraction of particulate NH_4_^+^ and non-NH_4_^+^ ions, and (**e**, **f**) gas-phase NH_3_ in Shijiazhuang (SJZ) and Beijing (BJ).

As shown in Fig. 1, the mass concentrations of NH_4_^+^ and non-NH_4_^+^ water-soluble ions in Shijiazhuang are 0.052–89.0 μg m^−3^ (median value of 7.5 μg m^−3^) and 0.9–241.2 μg m^−3^ (median value of 22.6 μg m^−3^), respectively, during the observation period (from March 15, 2018 to November 15, 2020). The corresponding values in Beijing are 0.06–47.7 μg m^−3^ (median value of 3.5 μg m^−3^ for NH_4_^+^) and 0.07–212.5 μg m^−3^ (median value of 9.1 μg m^−3^ for non-NH_4_^+^ ions). Compared with the previous observations, the air quality in both Shijiazhuang and Beijing has been significantly improved. For example, the mean concentration ±σ (standard deviation) of water-soluble NH_4_^+^ (10.5 ± 10.2 μg m^−3^) in Shijiazhuang is significantly lower than that (13.8 ± 13.6 μg m^−3^) measured from June 2014 to April 2016^37^. In Beijing, it is also lower (5.5 ± 5.6 μg m^−3^) than that measured from February to November 2017^38^ and even lower than the NH_4_^+^ concentration in nonrefractory PM_1_ (8.5 ± 7.9 μg m^−3^) from July 2011 to June 2012^39^.

The concentration of gas-phase NH_3_ in Shijiazhuang varies from 0.02 to 89.5 μg m^−3^ with a median value of 22.1 μg m^−3^, while it is from 1.0 to 43.2 μg m^−3^ with a median value of 15.6 μg m^−3^ in Beijing. The median value of NH_3_ concentrations in Beijing is comparable with previous observations carried out in Beijing and Xi’an^40^ but higher than that in Shanghai (4.6 μg m^−3^)^2^. It should be noted that the concentration of gas-phase NH_3_ shows a weak downwards trend in Shijiazhuang from March 2018 to November 2020 (Fig. 1e). The decreasing rate ±σ of NH_3_ is 2.3 ± 0.1 µg m^−3^ Yr^−1^ according to linear fitting. This value is almost the same as the decreasing rate (2.3 ± 0.4 µg m^−3^ Yr^−1^) fitted according to a combination of a sine function (due to seasonal variations of NH_3_ emissions^2,41^ and partition^42^) and a linear function (due to annual changes of NH_3_ emissions) for the probability-weighted NH_3_ concentrations based on a 2D Kernel density plot (Supplementary Fig. 1) by binning the dataset into 100×100 boxes to decrease the signal noise^43^.

As shown in Fig. 1c, however, the relative fraction of NH_4_^+^ in water-soluble ions is stable annually in Shijiazhuang. We even observe a slight upwards trend for the fraction of NH_4_^+^ in inorganic anions or nonrefractory PM_2.5_ measured by a Time-of-Flight Aerosol Chemical Speciation Monitor (ToF-ACSM, Aerodyne) in Beijing (Supplementary Fig. 2). This is in agreement with previous results that the NH_4_^+^ fractions increased significantly in Beijing from 2014 to 2019^44^. These results strongly imply that the partition equilibrium between gas-phase NH_3_ and particle-phase NH_4_^+^ interannually prefers particle-phase NH_4_^+^ in both Shijiazhuang and Beijing.

#### Kinetics

**Fig. 2:**
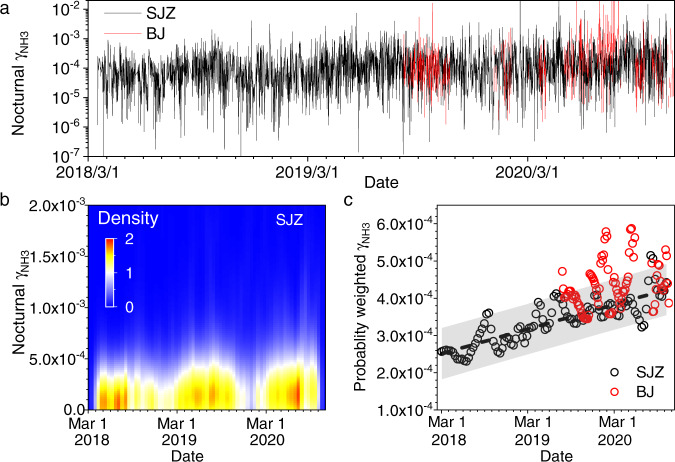
The variations of nocturnal uptake coefficient of NH_3_ (γ_NH3_). **a** Time series of nocturnal γ_NH3_ in Shijiazhuang (SJZ) and Beijing (BJ), **b** probability distribution of nocturnal γ_NH3_ in Shijiazhuang, and **c** probability-weighted nocturnal γ_NH3_ in Shijiazhuang and Beijing. The equation of the dashed line is γ_NH3_ = 2.50 × 10^−4^ + 6.58 × 10^−5^*t* (*R* = 0.83, *t*: year).

We calculate the nocturnal γ_NH3_ to decrease the influence of nucleation through gas-phase H_2_SO_4_ or HNO_3_ and NH_3_ as well as variations of advection and deposition on the γ_NH3_ calculations (the details can be seen in the SI). In Shijiazhuang, the derived nocturnal γ_NH3_ values are in the range of 4.02 × 10^−8^ to 8.02 × 10^−2^ with mean ±σ and median values of (1.13 ± 12.4) × 10^−4^ and 6.37 × 10^−5^ (Fig. 2), respectively. Although a variation in γ_NH3_ is discernible, no significant difference among different seasons is observed at the 0.05 level due to the noisy data, with mean ±σ γ_NH3_ values of (2.22 ± 6.87) × 10^−4^, (2.21 ± 4.20) × 10^−4^, (2.88 ± 7.70) × 10^−4^ and (2.52 ± 21.6) × 10^−4^ in spring, summer, autumn and winter, respectively, in Shijiazhuang (Supplementary Fig. 3). The γ_NH3_ values, which vary from 1.24 × 10^−6^ to 9.70 × 10^−2^ in Beijing, are comparable to those in Shijiazhuang during the same observation periods (Fig. 2a). The mean γ_NH3_ values reported here are significantly smaller than the γ_NH3_ on sulfuric acid (0.1–1)^29,30,32^, aqueous surfaces (~5 × 10^−3^–0.1) from pH in the range of 0–13^31^ and acidified secondary organic aerosol (~10^−3^–~10^−2^)^34^, while they are comparable with those on the surface of ice (5.3 ± 2.2 × 10^−4^)^27^ and sulfuric acid in the presence of organic gases (2 × 10^−4^–4 × 10^−3^)^23^.

γ_NH3_ shows an obvious upwards trend but with obvious variations in Fig. 2a. Figure 2b shows the probability distribution of nocturnal γ_NH3_ in Shijiazhuang, and Fig. 2c shows the probability-weighted nocturnal γ_NH3_ after the time series of γ_NH3_ is converted into a 2D kernel density plot. Three peaks with a high frequency of large γ_NH3_ occur in summer, while two minima are present in winter. The probability-weighted γ_NH3_, which statistically indicating weighted mean γ_NH3_, shows obvious seasonal variations and interannually increases with relative time in Shijiazhuang, e.g., γ_NH3_ = 2.50 × 10^−4^ + 6.58 × 10^−5^*t* (*R* = 0.83, *t*: year) or γ_NH3_ = 2.47 × 10^−4^–6.0 × 10^−5^sin(π(*t* + 1.99)/0.55) + 6.99 × 10^−5^*t* (*R* = 0.35, *t*: year). The probability-weighted γ_NH3_ values in Beijing are slightly higher than those in Shijiazhuang during the same period. They are more scattered due to the small dataset in Beijing than that in Shijiazhuang. However, a weak upwards trend is still observable in Beijing (Fig. 2c, *R* = 0.40) with a comparable slope of 5.15 × 10^−5^ Yr^−1^. These results mean that the γ_NH3_ increase by 6.24 ± 0.97 × 10^−5^ per year in the NCP, which further confirms the assumption that the formation of particle-phase NH_4_^+^ is preferred recently from the point of view of conversion kinetics.

### Driving force for the enhancement of γ_NH3_

A chemical reaction is usually sensitive to temperature. A low activation energy of 12.5 kJ mol^−1^ has been reported for the reaction between H_2_SO_4_ and NH_3_^30^. However, the decomposition of ammonium is more sensitive to temperature than the forward reaction^42^. Thus, a negative temperature dependence has been observed for the uptake of NH_3_ on the surface^31,42^. This means that the high temperature in summer should be unfavorable for the uptake of NH_3_ in the atmosphere.

**Fig. 3:**
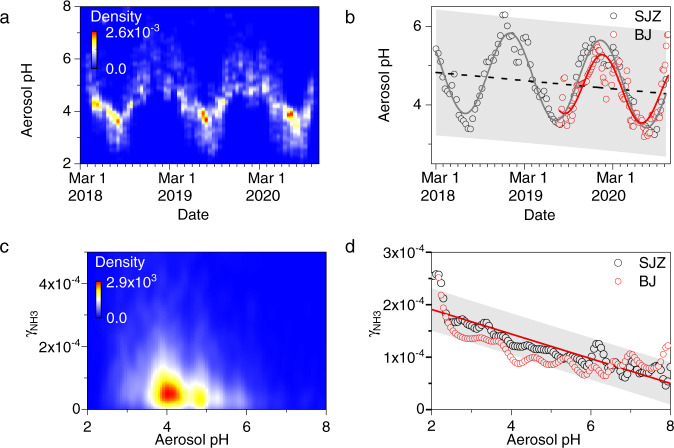
The aerosol pH and its correlation with nocturnal uptake coefficient of NH_3_ (γ_NH3_). **a** The probability distribution of the nocturnal aerosol pH in Shijiazhuang (SJZ), **b** the probability-weighted nocturnal aerosol pH in Shijiazhuang and Beijing (BJ), **c**, **d** the relationship between the nocturnal γ_NH3_ and the aerosol pH in Shijiazhuang and Beijing.

The formation of NH_4_^+^ includes the uptake of NH_3_ and the proton transfer reaction between protons (H^+^) and NH_3_. The positive dependence of γ_NH3_ on acidity for the uptake of NH_3_ on aqueous surfaces^31^ or organic aerosol surfaces^34^ has been reported previously. As shown in Fig. 3a, b, the aerosol pH values, which dominantly varies from 3.0 to 7.0 calculated using the ISORROPIA II model in Beijing and Shijiazhuang, are overall higher than those estimated in Nagoya, Japan (3.6–5.4)^45^, the cities in the Po Valley, Italy (1.5−4.5)^46^, and the northeastern United States (0.5–3)^47^, while they are comparable with those reported in northern China^44,48–51^. The relatively high aerosol pH in the NCP can be explained by the high concentrations of NH_3_ and dust in the atmosphere to neutralize sulfate and nitrate^52^.

Seasonal variations in aerosol pH are obvious (Fig. 3a, b), e.g., high aerosol pH in winter and low aerosol pH in summer. This is determined in a complicated manner by the composition of PM_2.5_, the air temperature, and the aerosol liquid water content (AWC) or RH^45,46,48,50,53^. The probability-weighted aerosol pH can be well fitted by a combination of a sine function which reflects the seasonal variation of aerosol pH, inversely depending on temperature^54^ and the ratio of SO_4_^2−^/NO_3_^−^^46^, and a linear function of time that reflecting interannual changes of aerosol pH, i.e., pH = 4.90–1.06sin(π(*t* − 0.58)/0.51) – 0.18*t* (*R* = 0.88, *t*: year) in Shijiazhuang and pH = 4.98–0.81 sin(π(*t* − 0.80)/0.44)–0.27*t* (*R* = 0.69, *t*: year) in Beijing. This means that the aerosol acidity ±σ increases by 0.23 ± 0.06 units per year. This is consistent with the interannual increase in γ_NH3_ (Fig. 2b). It should be noted that Song et al.^44^ observed a decrease in wintertime aerosol acidity from 2014 to 2019. In their work, the aerosol pH values were modeled using gaseous ammonia and chemical components of nonrefractory submicron particles (NR-PM_1_), including SO_4_^2−^, NO_3_^−^, NH_4_^+,^ and Cl^−^, as inputs. In this study, however, more inputs, including the measured gas-phase (NH_3_, HCl, and HNO_3_) and particle-phase (SO_4_^2−^, NO_3_^−^, Cl^−^, NH_4_^+^, K^+^, Na^+^, Ca^2+,^ and Mg^2+^) components, are accounted for. This should result in a more precise aerosol pH.

Figure 3c, d further show the relationship between γ_NH3_ and aerosol pH. The probability-weighted γ_NH3_ and aerosol pH show a linear correlation, i.e., γ_NH3_ = 2.38 × 10^−4^–2.35 × 10^−5^pH (*R* = 0.90, in Shijiazhuang) and γ_NH3_ = 2.06 × 10^−4^–1.90 × 10^−5^pH (*R* = 0.63, in Beijing). This is consistent with the positive correlation between the *k*_het_ of NH_3_ and the acidity of aerosols, expressed by the H^+^/NH_4_^+^ mole ratio^55^. The negative correlation between γ_NH3_ and aerosol pH can be well explained in light of the linear free energy relationship for a reaction series^56^, which is also observed in our previous work on the uptake of amines by ammonium salts^57^ and organic acids^58^. In addition, these results are in agreement with the formation of NH_4_NO_3,_ which is HNO_3_ sensitive according to the partition of nitric acid and ammonia to the aerosol phase^59^ (Supplementary Fig. 4).

Silvern et al.^34^ found that aerosols are becoming more acidic even as SO_2_ emissions decrease and ammonia emissions remain constant in the United States. They proposed that sulfate particles are increasingly coated by organic matter (OM), retarding the uptake of ammonia similar to that observed in chamber studies^23^. However, we do not observe an increase in the OM/sulfate ratio during our observations, as shown in Supplementary Fig. 5. In addition, the OM/sulfate ratios in Beijing are higher than those in Shijiazhuang, while both the aerosol pH and the γ_NH3_ are comparable in these two locations. Recent studies proposed that conversion process from NOx to NO_3_^−^ could affect the conversion process from NH_3_ to NH_4_^+^^60^. Although a seasonal variation of the NOR is observable, i.e., higher NOR values are observed in winter compared to summer, the probability-weighted nitrogen oxidation ratio (NOR) is annually stable during our observations in Shijiazhuang (Supplementary Fig. 6), unlike the observed γ_NH3_ or pH. These results mean that the annual increase in aerosol pH should not be mainly regulated by the organic film in aerosols since inorganic and organic compounds are expected to reside in separate phases in Beijing^44^ or variations in NOR.

**Fig. 4:**
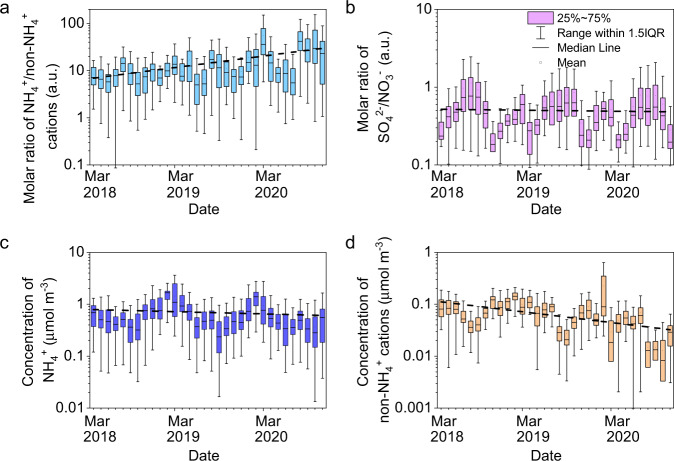
The variations of NH_4_^+^, non-NH_4_^+^ cations, SO_4_^2−^ and NO_3_^−^. **a**, **b** The monthly mean values of the ratios of NH_4_^+^/other cations and SO_4_^2−^/NO_3_^−^; **c**, **b** the mass concentrations of NH_4_^+^ and other cations in Shijiazhuang. The dashed lines are a linear fit of the monthly mean values.

The composition should be the important factor affecting the annual increase in aerosol acidity^45–47,53^ because we do not observe interannual variations in AWC and temperature (Supplementary Fig. 7). SO_4_^2−^, NO_3_^−^, NH_4_^+^, and other cations (K^+^, Na^+^, Ca^2+^, Mg^2+^) are the major components that influence the aerosol pH. Figure 4 shows the monthly mean ratios of NH_4_^+^/other cations, SO_4_^2−^/NO_3_^−^ as well as the corresponding mass concentrations. The ratio of SO_4_^2−^/NO_3_^−^ shows a weak decreasing trend. This should not result in the interannual enhancement of aerosol acidity because it is positively correlated with the ratio of SO_4_^2−^/NO_3_^−^^46^. In contrast, an upwards trend for the ratio of NH_4_^+^/other cations is observable in Fig. 4a. Additionally, NH_4_^+^ shows a weak down or relatively stable trend, while a more obvious reduction of other cations can be seen in Fig. 4d. Ca^2+^ contributes 55.1 ± 20.3% to the total mass of these alkali and alkali earth metals. It is well known that the basicity of NH_4_^+^ is significantly weaker than that of alkali and alkali earth metals. In addition, Ca^2+^ can react with SO_4_^2−^ to form insoluble CaSO_4_, which is out of the aerosol aqueous phase, subsequently reducing the aqueous SO_4_^2−^ and thus the particle acidity^61^. The decrease in Ca^2+^ concentration thus inevitably leads to a weakened neutralization or precipitation ability to acids. In previous work, it has also been found that mineral dust plays an important role in decreasing aerosol pH^53,61–63^. These results strongly imply that the interannual reduction in aerosol pH should be dominated by the decline in alkali and alkali earth metals^64^ in both Shijiazhuang and Beijing.

To further confirm the importance of alkali and alkali earth metals to aerosol pH, machine learning has been performed using a random forest model^65^, which is a highly accurate classification algorithm. Twenty-three meteorological and chemical parameters are used as inputs to investigate their effect on aerosol pH. Ca^2+^ is the third most important factor after temperature and RH to aerosol pH (Supplementary Fig. 8). Mg^2+^, K^+^, and Na^+^ also significantly contribute to aerosol pH. These results are consistent with the decreased fugitive dust emissions in the North China Plain due to the intensive regulation of dust emissions^66–68^. It should be pointed out that organics are not considered when we calculate aerosol pH. If the fraction of organic acids increases annually during our observations, it should also lead to an upwards trend in aerosol acidity. The mass fraction of m/z 44 (*f*_44_), which is usually an indicator of carboxylic acids in OA^69^, is interannually stable, although *f*_44_ varied seasonally in Beijing (Supplementary Fig. 9). Therefore, we can conclude that the interannually elevated aerosol acidity should not be determined by the variations in organic acid content although aerosol acidity might be underestimated by the ISORROPIA II model that cannot consider organic acids.

**Fig. 5:**
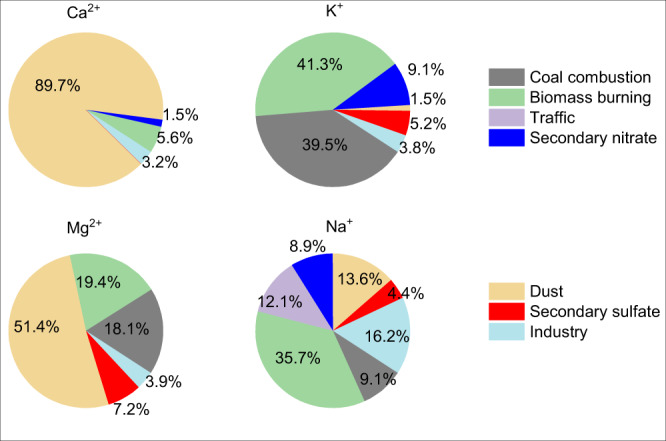
The sources of soluble alkali metals in PM_2.5_. The relative mean contribution from coal combustion, biomass burning, traffic, secondary nitrate, dust, secondary sulfate and industry sources to the mass concentrations of Ca^2+^, Mg^2+^, K^+^ and Na^+^ ions.

Seven sources of PM_2.5_, including coal combustion, biomass burning, traffic, secondary nitrate, dust, secondary sulfate, and industry, have been identified in Shijiazhuang using the measured concentrations of inorganic ions, OC, EC, and heavy metals. The Q_true_/Q_exp_ is 4.3 for the seven-factors solution. This value corresponds to a relatively stable variation of the d(Q_true_/Q_exp_)/dN (Supplementary Fig. 10). Supplementary Figs. 11 and 12 show their source profiles and the time series of hourly mean mass concentrations. Coal combustion is characterized by high loadings of OC, EC, and heavy metals; biomass burning is characterized by high concentrations of Cl and K and moderate OC and EC; traffic shows a high fraction of Zn and moderate concentrations of EC and OC; dust sources show high fractions and concentrations of Ca^2+^, Mg^2+^, Al and Ti; secondary nitrate and secondary sulfate are characterized by high concentrations of nitrate and sulfate, respectively; industrial sources are connected with high concentrations of Fe, Co, Mn, etc. These sources are well supported by the diurnal and monthly variations (Supplementary Fig. 13). The mean ± σ contributions of these 7 sources to the PM_2.5_ mass concentration during the observations in Shijiazhuang are 15.3 ± 7.2, 10.6 ± 12.5, 14.6 ± 8.1, 12.3 ± 10.1, 16.9 ± 15.5, 14.6 ± 10.0, and 15.6 ± 11.9%, respectively. As shown in Fig. 5, dust explains 89.7, 51.4, and 13.6% of the soluble Ca^2+^, Mg^2+^, and Na^+^, respectively. In addition, the mass concentration of dust decreased obviously (Supplementary Fig. 12), with an annual decrease rate of 0.90 ± 0.03 µg m^−3^ yr^−1^. The contribution of dust to PM_2.5_ mass concentration also shows a prominent decrease (10.8 ± 2.5% yr^−1^) as shown in Supplementary Fig. 14. This means that the reduction in dust emissions mainly regulates the increase in aerosol acidity observed in Fig. 3.

## Discussion

Liggio et al.^23^ found that competition for uptake between NH_3_ and organic gases can block the surface of sulfuric acid aerosols from incoming NH_3_, subsequently slowing the approach to thermodynamic equilibrium. This was further validated by modeling studies that explained the low ammonium-sulfate ratio (1.04 ± 0.21 mol mol^−1^) in the eastern United States, even though ammonia was in large excess^70^. The uptake of NH_3_ by sulfuric acid aerosols can also be retarded by precoated *n*-hexadecanol^35^ and *n*-hexadecane^36^. However, as shown in Supplementary Fig. 5, the OM/sulfate ratio is relatively stable during our observations. In addition, the OM/sulfate ratio in Beijing is obviously higher than that in Shijiazhuang during the same observational period, while the γ_NH3_ is comparable between these two locations. This means that the observed interannually increase in γ_NH3_ cannot be explained by the changes in organic coating. In the atmosphere, the mixing state of aerosols is more complicated than that of model aerosols^71^ and subsequently has a complex influence on the reaction kinetics of trace gases, similar to other reaction systems^72,73^. Thus, we ascribe the relatively small γ_NH3_ in this work to the matrix effect of ambient aerosols. On the other hand, Kuwata and Martin^74^ showed that the amount and rate of ammonia uptake by secondary organic aerosol depends strongly on RH due to both thermodynamics and the influence of particle viscosity on uptake kinetics. We also observe an obvious increase of the probability-weighted γ_NH3_ at high RH values, in particular, over 80% of RH (Supplementary Fig. 15). However, the interannual changes of RH is not observed. This means that variations of RH might partially contribute to the wide range of the γ_NH3_ (Fig. 2a), while it cannot statistically explain the interannual increase of the γ_NH3_. It should be noted that the probability-weighted γ_NH3_ varies from 2.31 × 10^−4^ to 5.87 × 10^−4^ in the NCP (Fig. 2c). It is smaller than the measured γ_NH3_ on model particles, but close to that (5 × 10^−4^) utilized in a modeling study^70^. Further studies are required for understanding the impacts of γ_NH3_ values on atmospheric chemistry including nitrogen cycle and PM_2.5_ mass loading in the future.

NH_3_ is mainly neutralized by H_2_SO_4_ and HNO_3_. In the daytime, the gas-phase concentration of H_2_SO_4_ is estimated to be from 6.35 × 10^5^ to 5.49 × 10^6^ molecules cm^−3^ based on a proxy method^75^ in Shijiazhuang. The condensed H_2_SO_4_ only contributes (0.14 ± 0.17)% to particle-phase NH_4_^+^ and (0.70 ± 1.91)% to particle-phase SO_4_^2−^. The mean ±σ γ_NH3_ will be underestimated by (2.0 ± 6.4)% if the condensation of H_2_SO_4_ is not accounted for (Supplementary Fig. 16). At night, the contribution of gas-phase H_2_SO_4_ can be ruled out because it is mainly produced from photochemical oxidation of SO_2_ in the daytime, and its nighttime concentration is very low^75^. However, gas-phase HNO_3_ might be released through the hydrolysis of heterogeneous hydrolysis of N_2_O_5_ at night^76–78^. Although nucleation between HNO_3_ and NH_3_ at low temperatures and high nitric acid concentrations has been observed in chamber studies^79^, this process has not yet been validated in any field measurements. According to the chamber results, particle growth via nucleation between HNO_3_ and NH_3_ is active when the product of HNO_3_ and NH_3_ concentrations (*c*_HNO3×_*c*_NH3_) is higher than 5 × 10^5^ pptv^2^ and the temperature is lower than 278.15 K^79^. We have checked the nocturnal dataset with PM_2.5_ concentrations lower than 35 μg m^−3^ and a duration longer than 3 h during our observations. Forty-two events have been picked out with *c*_HNO3×_*c*_NH3_ larger than 5 × 10^5^ pptv^2^ (*c*_HNO3_ ± σ: 0.19 ± 0.09 ppbv; *c*_NH3_ ± σ: 14.1 ± 10.5 ppbv). However, we do not observe any nucleation or growth event based on the particle size spectrum even though the PM_2.5_ mass concentration (20.2 ± 8.9 μg m^−3^) and the temperature (273.6 ± 3.1 K) are low enough. Therefore, we postulate that the contribution of nucleation between HNO_3_ and NH_3_ to particle-phase NH_4_^+^ should be negligible.

It should be noted that the concentrations of ions vary from 3.2 to 30.7 mol L^−1,^ and the ionic strength in the aerosol liquid phase vary from 1.8 to 23.2 mol L^−1^ during our observations (Supplementary Fig. 17a). The ISORROPIA II model thus usually overestimates aerosol acidity because it assumes a unity of activity coefficient (γ_H+_)^80^. We have calculated the activity coefficient, which is significantly lower than unity (Supplementary Fig. 17a), according to the method derived by Glueckauf for concentrated electrolyte solutions^81,82^. As shown in Supplementary Fig. 17b, the aerosol pH_F_ (γ_H+_ = 1.0) values are well correlated with the aerosol pH_γ_ values (slope = 1.09, R = 0.99), which accounted for the γ_H+_. However, the aerosol pH_F_ values are lower at 1.27 units (the intercept) than the aerosol pH_γ_ values. This is in agreement with a recent study that found that the pH_F_ calculated by ISORROPIA II was ~1 unit lower than the pH_γ_ calculated by models in which the activity coefficient was accounted for^80^. However, as shown in Supplementary Fig. 17c, d, the pH_γ_ shows similar seasonal and interannual variations as the pH_F_. This means that the dependence of γ_NH3_ and aerosol pH (Fig. 3d) should not be affected by the uncertainty of pH from the activity coefficient of H^+^. This does so for the relationship between aerosol pH and the content of alkali and alkali earth elements (Fig. 4).

SO_2_ emissions have decreased significantly in Asia since 2005, while NOx emissions show a lower decline rate^83^. This leads to a decrease in the ratio of SO_4_^2−^/NO_3_^−^ in PM_2.5_^20,84^, as observed in the United States^85^. It is well recognized that the concentration of SO_4_^2−^^45,64,86^ or the ratio of SO_4_^2−^/NO_3_^−^^87^ can significantly regulate aerosol acidity, i.e., a higher SO_4_^2−^/NO_3_^−^ traditionally corresponds to a stronger aerosol acidity^45^. Thus, a decrease in aerosol acidity is expected in many regions, including East Asia, because of the shifting inorganic aerosol composition from ammonium sulfate to ammonium nitrate^85^. However, an increase in aerosol acidity is observed in the typical cities of the NCP (Shijiazhuang and Beijing) in this work, although the ratio of SO_4_^2−^/NO_3_^−^ decreases slightly. A machine learning study and source apportionment have confirmed that a significant reduction in alkali and alkali earth metals compensates for the increase in aerosol pH resulting from the decreased sulfate content in PM_2.5_. The increased aerosol pH favors the uptake of NH_3_ in both Shijiazhuang and Beijing, thus leading to a constant or slightly increased NH_4_^+^ fraction in PM_2.5,_ as observed in this work. NH_4_^+^ can further affect aerosol acidity via both neutralizing^80,88^ and buffering effects^50,85^. Aerosol acidity plays an important role in not only secondary organic aerosol formation, as also recognized in previous studies^89^ but also secondary inorganic aerosol formation, as observed in this study, and subsequently aerosol mass loadings and chemical composition. Besides ammonium formation, the formation of sulfate and nitrate is also sensitive to aerosol pH, e.g., a high aerosol pH is in favor of heterogeneous conversion from SO_2_ to sulfate^90,91^, the partition of semi-volatile nitrate^59,63^, and uptake of N_2_O_5_ to form nitrate^92^. In addition, strong aerosol acidity favors the dissolution of toxic heavy metals^93^ and thus the toxicity of aerosols. In developing regions, the regulation of dust emissions is one of the most effective administration strategies targeting PM_2.5_ pollution. Our results mean that more attention should be given to the indirect effects of these strategies on air quality and human health in the future.

## Methods

### Field observations

Observations were carried out at Aerosol and Haze Laboratory, Beijing University of Chemical Technology (AHL/BUCT Station, Lat. 39°56ʹ31ʺ and Lon. 116°17ʹ52ʺ) from August 8, 2019, to November 15, 2020, and Hebei Atmospheric Supersite, which is in Shijiazhuang University (HAS/SJZ, Lat. 38.0281°, and Lon. 114.6070°) from March 15, 2018, to November 15, 2020. The details about the observation stations have been described in previous work^94–96^. Briefly, both were on the rooftop of the corresponding main teaching building (5 floors, ~18 m, and ~23 m, respectively, above the surface for AHL/BUCT and HAS/SJZ stations) and are typical urban observation stations surrounded by traffic and residential emissions. The locations of the stations are shown in Supplementary Fig. 18.

The instruments in this study are shown in Supplementary Table 1. Briefly, the mass concentrations of PM_2.5_ were measured using a Taper Element Oscillating Microbalance (TEOM 1405-DF, ThermoFisher) at the AHL/BUCT station and a Beta Attenuation Mass Monitor (BAM-1020, Met One Instruments) at the HAS/SJZ station. Water-soluble ions (Cl^−^, NO_3_^−^, SO_4_^2−^, Na^+^, NH_4_^+^, K^+^, Mg^2+^, and Ca^2+^) in PM_2.5_ and gas pollutants (HCl, HONO, HNO_3_, SO_2,_ and NH_3_) were measured using an analyzer for Monitoring AeRrosols and Gases in ambient Air (MARGA, 2060R at the AHL/BUCT station and 2080 at the HAS/SJZ station, Metronhm Process Analytics) with 1 h of time resolution. Trace gases, including NO_x_, SO_2_, CO, and O_3,_ were measured with the corresponding analyzer (Thermo Scientific, 42i, 43i, 48i, and 49i). A Particle Size Magnifier (PSM, Airmodus), a Neutral cluster & Air Ion Specter (NAIS, Airel Ltd), a Differential Mobility Particle Sizer (DMPS, Custom made, University of Helsinki), and an Aerodynamic Particle Sizer (APS 3321, TSI) were deployed to measure the particle size distribution from 1 nm to 10 μm at the AHL/BUCT station, while a Scanning Mobility Particle Sizer (SMPS, TSI) consisting of a Differential Mobility Analyzer (DMA 3938, TSI) and a Condensation Particle Counter (CPC 3776, TSI) and an APS (3321, TSI) were available at the HAS/SJZ station. The bulk composition, including chloride, nitrate, sulfate, ammonium, and organics of nonrefractory PM_2.5_ (NR-PM_2.5_), was measured with a time-of-flight aerosol chemical speciation monitor (ToF-ACSM, Aerodyne) at the AHL/BUCT station. Organic carbon (OC) and element carbon (EC) were measured using the National Institute for Occupational Safety and Health (NIOSH, 5409) protocol by OC/EC analyzers (Model 4, Sunset) at both stations. The concentrations of heavy metals were measured using an X-ray fluorescence Atmospheric Heavy Metal Online Analyzer (EHM-X100, Skyray Instruments). Meteorological parameters, including temperature, pressure, relative humidity (RH), wind speed, and direction, were measured using weather stations (ASW310 at the AHL/BUCT station and WXT 520 at the HAS/SJZ station, Vaisala).

The MARGAs were externally calibrated using anionic solutions (Cl^−^, Br^−^, NO_3_^−^, SO_4_^2−^) and cationic solutions (Li^+^, Na^+^, K^+^, Mg^2+,^ and Ca^2+^) monthly. Internal calibration was also carried out hourly using LiBr standard solutions. The detection limits of Cl^−^, NO_3_^−^, SO_4_^2−^, Na^+^, NH_4_^+^, K^+^, Mg^2+^, and Ca^2+^ were 0.01, 0.05, 0.04, 0.05, 0.05, 0.09, 0.06 and 0.09 μg m^−3^, respectively. All the instruments for trace gas measurements were calibrated weekly using the corresponding standard gases. The detection limits were 0.05, 0.05, 40, and 0.5 ppbv for NO_x_, SO_2_, CO, and O_3_, respectively. External calibration was performed for the OC/EC analyzers biweekly using sucrose solutions. The heavy metal analyzer was calibrated with external standards.

### Calculations of γ_NH3_ and aerosol pH

NH_3_ mainly reacts with H_2_SO_4_ and HNO_3_ in the atmosphere. The reaction is a prototypical acid-base neutralization, i.e., proton transfer reaction, which occurs instantaneously in aqueous solutions. Although nucleation through H_2_SO_4_ and NH_3_ contributes to new particle formation^97^, this process usually cannot explain the high nucleation rates observed in different environments^98,99^ and the growth of ammonium salts. Nucleation through HNO_3_ and NH_3_ in the gas phase has not yet been validated in ambient air^70^, although it has been observed in a laboratory study at low temperature and high HNO_3_ or NH_3_ concentrations^79^. These results mean that NH_3_ should mainly contribute to particle growth via heterogeneous uptake. Therefore, the rate constant for conversion from NH_3_ to NH_4_^+^ through a heterogeneous reaction can be derived in the same way as calculating the conversion rate constant of NO_2_ to HONO^96,100,101^. As shown in Eq. (1),

1 $${k}_{{het}}=\frac{2({c}_{{{NH}}_{4}^{+},{t}_{2}}-{c}_{{{NH}}_{4}^{+},{t}_{1}})}{({c}_{{{NH}}_{3},{t}_{2}}+{c}_{{{NH}}_{3},{t}_{1}})({t}_{2}-{t}_{1})}$$

where *k*_het_ is the quasi-first-order reaction rate constant for heterogeneous conversion (s^−1^) and *c*_NH4+,ti_ and *c*_NH3_,_ti_ are the concentrations of the particle-phase concentrations of NH_4_^+^ and NH_3_ at a given time *t*_i_ (ppbv or μg m^−3^). The *k*_het_ was calculated when *c*_NH4+_ increases, while *c*_NH3_ decreases assuming a constant emission rate from *t*_1_ to *t*_2_ (within 1 h). The nocturnal *k*_het_ values are 1.3 ± 2.1 × 10^−5 ^s^−1^ and 1.2 ± 2.4 × 10^−5 ^s^−1^, respectively, in Shijiazhuang and Beijing. They are in the range of the *k*_het_ (4.0 × 10^−6 ^s^−1^–4.1 × 10^−4^) by measuring the conversion of NH_3_ to NH_4_^+^ as the air mass traveled between three successive distant sampling points in rural England^55^. Then, the nighttime uptake coefficient of NH_3_ (γ_NH3_) was calculated according to

2 $${\gamma }_{{{NH}}_{3}}=\frac{4{k}_{{het}}}{S\omega }=\frac{4{k}_{{het}}}{S\sqrt{\frac{8{RT}}{\pi M}}}$$

where *S* is the surface-to-volume ratio of aerosols (m^−1^) and is measured using these instruments for particle size distribution, ω is the mean velocity of NH_3_ molecules, *R* is the ideal gas constant, *T* is the air temperature (K), and *M* is the molecular weight of NH_3_ (kg mol^−1^). The uncertainty for calculating the γ_NH3_ is discussed in the SI (Supplementary Figs. 19 and 20 and Supplementary Table 2).

The aerosol water content (AWC) and pH were estimated by combining an aerosol thermodynamic model and the measured particle composition with T and RH, assuming that the aerosol system was in equilibrium. The ISORROPIA II model, which is widely used in aerosol pH estimation^48,49,53,102^, was used in the “forward” mode assuming that the particles were in the “metastable” phase state to predict the concentration of H^+^ per volume of air (*c*_H+_, μg m^−3^) and the concentration of AWC (μg m^−3^)^46,54^. The pH is then calculated according to

3 $${{{{{\rm{pH}}}}}}=-{{{{{{\rm{log }}}}}}}_{10}\frac{1000{\gamma }_{{H}^{+}}{c}_{{H}^{+}}}{{AWC}}$$

where γ_H+_ is the activity coefficient of H^+^ (assumed to be 1.0)^46,54^. Under such an assumption, the calculated pH is pH_F_ indeed based on only the free-H^+^ molality^80^. Both the inorganic species and part of the organic species in particles are hygroscopic. Similar to previous work^48,49,53,102^, aerosol pH and AWC were predicted with only inorganic species because pH prediction was not highly sensitive to water uptake by organic species^53^, and the mass fraction of organic matter-induced particle water accounted for only 5% of the total AWC in Beijing^103^. AWC becomes very small when RH is low, subsequently introducing considerable uncertainty to aerosol pH^47,53,80^. Thus, we only calculated the aerosol pH for data with RH higher than 35%^80^.

### Identification of factors affecting aerosol pH

A random forest model (RF) was used to identify the crucial factors that affect aerosol pH. We used the classification and regression tree (CART) decision tree as the base learner for integrated learning. According to the bagging algorithm^65^, several independent and identically distributed training subsets are randomly obtained from the training set, and numerous decision trees are constructed according to the training subsets. We used the Gini coefficients as the criteria for node feature selection. Supplementary Fig. 21 shows the error loss during the training of the model. It can be seen that no underfitting or overfitting occurred in this model. In addition, the aerosol pH predicted by the RF model has a satisfactory correlation coefficient (*R* = 0.95) with that calculated using the ISORRPIA-II model. The relative standard deviation (RSD) is 4.61%.

Source apportionment of PM_2.5_ in Shijiazhuang was performed using a receptor model (positive matrix factor, PMF, EPA 5.0). The model was fed with the hourly mean concentrations (*c*_ij_) and the uncertainties (*S*_ij_) of species, including water-soluble ions, OC, EC, and heavy metals. The *S*_ij_ for each species was calculated using measurement uncertainties (MU%) and method detection limits (MDL)^104^. If *c*_ij_ ≤ MDL,

4 $${S}_{{ij}}=\frac{5}{6}\times {MDL}$$

Unless

5 $${S}_{{ij}}=\sqrt{{\left({MU}\times {c}_{{ij}}\right)}^{2}+{(0.5{MDL})}^{2}}$$

The dataset from May 13, 2019, to November 15, 2020, was used for source apportionment because of the availability of the heavy metal analyzer.

## Supplementary information

Suplementary information

## Data Availability

All data are available in the main paper and the Supplementary Information files. Source data files are provided for Figs. 1–5. Source data are provided in this paper. Source data are provided with this paper.

## Source data

Source Data
