# Supplementary material for: Dust emission reduction enhanced gas-to-particle conversion of ammonia in the North China Plain
Source: Nat Commun. 2022 Nov 12;13:6887. doi: 10.1038/s41467-022-34733-4 (PMC9653376; doi:10.1038/s41467-022-34733-4)
Supplement: Supplementary file 1 — Suplementary information [file 41467_2022_34733_MOESM1_ESM.docx]

Supplementary Information for

**Dust emission reduction enhanced gas-to-particle conversion of ammonia in the North China Plain**

Yongchun Liu^1*^, Junlei Zhan^1^, Feixue Zheng^1^, Boying Song^1^, Yusheng Zhang^1^, Wei Ma^1^, Chenjie Hua^1^, Jiali Xie^1^, Xiaolei Bao^2,3*^, Chao Yan^4,5^, Federico Bianchi^4^, Tuukka Petäjä^1,4,5^, Aijun Ding^5^, Yu Song^6^, Hong He^7^, Markku Kulmala^1,4^

1. Aerosol and Haze Laboratory, Advanced Innovation Center for Soft Matter Science and Engineering, Beijing University of Chemical Technology, Beijing 100029, China

^2^ Hebei Technological Innovation Center for Volatile Organic Compounds Detection and Treatment in Chemical Industry, Hebei Chemical & Pharmaceutical College, Shijiazhuang 050026, China

^3^ Hebei Provincial Academy of Environmental Sciences, Shijiazhuang 050037, China

^4^ Institute for Atmospheric and Earth System Research, Faculty of Science, University of Helsinki, Helsinki 00014, Finland

^5^ Joint International Research Laboratory of Atmospheric and Earth System Sciences, School of Atmospheric Sciences, Nanjing University, Nanjing 210023, China

^6^ State Key Joint Laboratory of Environmental Simulation and Pollution Control, College of Environmental Sciences and Engineering, Peking University, Beijing 100871, China

^7^ Research Center for Eco-Environmental Sciences, Chinese Academy of Sciences, Beijing 100085, China

* Corresponding authors. Email: liuyc@buct.edu.cn (Y. L.); bxl5@163.com (X. B.)

**Uncertainty of uptake coefficients.**

When calculating the first-order reaction rate constant according to Eq. (1) in the manuscript, we use only pairs of time points for which particle phase ammonium increases and gas phase ammonia decreases assuming a constant emission rate between subsequent hours. By doing so, we assumed a steady state, which is usually taken when one deriving uptake kinetics of trace gases on ambient particles based on field observations ^1, 2, 3, 4, 5^.

According to mass conservation, the concentration of any air pollutant should be described by,

$\frac{dc}{dt}=E+T-R-D$ (1)

where, *E*, *T*, *R*, and *D* denote the emission rate, transport rate, reaction rate, and deposition rate, respectively. We assumed that the values of *E*, *T*, and *D* are constant within a short time, i.e., one hour during our calculations. Thus, the net uptake is assumed to be the main factor driving its concentration changes, and a decrease in NH_3_ concentration is also assumed when we calculating the γ_NH3_. Although this is an ideal condition, we think the assumption should be reasonable because of the following reasons.

i) Non-agricultural emissions from fossil fuel combustion, waste, and livestock are the dominant sources of NH_3_ in the Urban Environment of the NCP ^6, 7^. It is reasonable to assume a stable emission rate from these sources within a short period although a real-time emission inventory of NH_3_ is unavailable at the present. ii) Relatively low nocturnal wind speeds were observed during our observations. The median and mean wind speeds were 1.3 and 1.5 m s^-1^, respectively, at a height of ~23 m in Shijiazhuang (Supplementary Figure 19A). This means that the concentration changes of air pollutants should be mainly related to local air mass within one hour of the time interval. It should be noted that the distribution of nocturnal NH_3_ concentrations was highly overlapped with that of NH_4_^+^ concentrations (Supplementary Figure 19B and C). This means that NH_3_ and NH_4_^+^ will be in the same air mass even if the wind direction changes. iii) We checked the hourly vertical profile of temperature from 1000 to 10 hPa in Shijiazhuang using the data from the Copernicus Climate Change Service (C3S) Climate Data Store (CDS, https://www.copernicus.eu/en). There were 1673 γ_NH3_ corresponding to temperature inversion out of the total data points (5182) in Fig. 2A. Supplementary Figure 20A shows the nocturnal γ_NH3_ corresponding to temperature inversion and non-temperature inversion in Shijiazhuang. The γ_NH3_ values when temperature inversion occurs ((1.76±3.94)×10^-4^) are smaller than the counterpart ((2.65±14.8)×10^-4^). The overestimation is ~34% if all the nocturnal data points are taken into consideration ((2.36±12.4)×10^-4^) when compared with those constrained by temperature inversion. iv) A stable deposition rate of NH_3_ was reported at night ^8^ because a temperature inversion usually occurred, in particular, from 00:00 to 07:00. We further classified the nocturnal γ_NH3_ into two subsets, i.e., 00:00-07:00 (subset-I) and 18:00-23:00 (subset-II). As shown in Supplementary Figure 20B, the γ_NH3_ values in subset-II are higher than those in subset-I. An overestimation is 22% for all the nocturnal γ_NH3_ when compared with the subset-I (Supplementary Table 2). Supplementary Figure 20C further compares the nocturnal γ_NH3_ constrained by a decrease in NH_3_ concentration with the rest data points constrained by a constant or increase in NH_3_ concentration. Overall, the constrained γ_NH3_ values are larger than the rest values. The mean of the constrained γ_NH3_ ((2.36±12.4)×10^-4^) is also larger than the counterpart ((1.79±5.87)×10^-4^) (Supplementary Table 2). All the nocturnal γ_NH3_ are overestimated by ~24% compared with the non-constrained data.

In summary, the implicit assumption of the calculation should inevitably lead to uncertainties in the γ_NH3_. A random combination of other factors might be one of the reasons why the calculated γ_NH3_ varied greatly (Fig. 2A). This will increase the noise of the data rather than the trend when the size of the data set is big enough. It should be pointed out that there are 5182 data points in Shijiazhuang (Fig. 2A) and 3898 data points in Fig. 3A. We think the size of the data (2.5 years) should be enough to do a statistical analysis. It is well supported by almost the same increase trends of γ_NH3_ among different subsets of the data as shown in Supplementary Figure 20. On the other hand, we introduced a probability-weighted γ_NH3_ and aerosol pH to discuss their trends and correlations. This method can reduce the influence of some outliers. It should be pointed out that the median lifetime of NH_3_ via heterogeneous uptake is around 1-2 days. This is reasonable when compared with the atmospheric lifetime of NH_3_ (from hours to days) ^9^. Therefore, we can conclude that the uncertainty of γ_NH3_ should not affect the main conclusions of this study.

**Supplementary Table 1**. Instrumentations used in the AHL/BUCT and HAS/SJZ stations

| Parameters | Instruments | | |
| --- | --- | --- | --- |
|  | HAS/SJZ station | AHL/BUCT station | |
| PM_2.5_ mass | Beta Attenuation Mass Monitor (BAM-1020, Met One Instruments) | Taper Element Oscillating Microbalance (TEOM, 1405-DF, Thermo Fisher) | |
| Water-soluble ions | Monitoring AeRrosols and Gases in ambient Air (MARGA 2080, Metronhm Process Analytics) | Monitoring AeRrosols and Gases in ambient Air (MARGA 2060R, Metronhm Process Analytics) | |
| Non-refractory PM_2.5_ composition | - | Time-of-Flight Aerosol Chemical Speciation Monitor (ToF-ACSM, Aerodyne) | |
| OC/EC | OC/EC Analyzer (Model 4, Sunset) | | |
| Heavy metals | Atmospheric Heavy Metal Online Analyzer (EHM-X100, Skyray Instruments) | | - |
| Particle size distribution | Scanning Mobility Particle Sizer (SMPS 3938, TSI), Aerodynamic Particle Sizer (APS 3321, TSI) | Particle Size Magnifier (PSM, Airmodus), Neutral cluster & Air Ion Specter (NAIS, Airel Ltd)), Differential Mobility Particle Sizer (DMPS, University of Helsinki), Aerodynamic Particle Sizer (APS 3321, TSI) | |
| Weather conditions | Weather station (WXT 520, Vaisala) | Weather station (AWS310, Vaisala) | |
| Trace gases | NOx, SO_2_, CO and O_3_ analyzer (42i, 43i, 48i and 49i, Thermo Scientific) | | |

**Supplementary Table 2.** The γ_NH3_ calculated with different constraint conditions in Shijiazhuang.

| Constraint conditions | | γ_NH3_ | | Median lifetimes (h) |
| --- | --- | --- | --- | --- |
|  |  | Mean±SD | Median |  |
| Decrease in NH_3_ concentration | all | (2.36±12.4)×10^-4^ | 9.97×10^-5^ | 34.6 |
|  | 00:00-07:00 | (1.93±15.2)×10^-4^ | 8.35×10^-5^ | 37.9 |
|  | 18:00-23:00 | (2.91±7.59)×10^-4^ | 1.29×10^-4^ | 30.2 |
|  | Temperature inversion | (1.76±3.94)×10^-4^ | 7.71×10^-5^ | 33.9 |
|  | Non-Temperature inversion | (2.65±14.8)×10^-4^ | 1.12×10^-4^ | 35.0 |
| Constant or increase in NH_3_ concentration | | (1.79±5.87)×10^-4^ | 6.69×10^-5^ | 48.2 |
| Unconstrained | | (2.21±11.1)×10^-4^ | 8.96×10^-5^ | 37.7 |

**Supplementary Fig. 1**. **The variation of gas-phase NH_3_ concentration.** (a) The probability distribution of NH_3_ concentration in Shijiazhuang and (b) the probability-weighted NH_3_ concentrations in Shijiazhuang and Beijing. The equation of the dash line is: *y* = 26.3 - 2.28*t* (*R*=0.14), while the equation of the solid line is: *y* = 25.7 - 4.0sin(π(*t*+0.53)/0.52) – 2.26*t* (*R*=0.51).

**Supplementary Fig. 2**. **The trend of particulate NH_4_^+^ in Beijing**. (a) The mass fraction of NH_4_^+^ in inorganic anions measured using an ACSM and (b) the fraction of NH_4_^+^ in nonrefractory PM_2.5_ in Beijing.

**Supplementary Fig. 3. The seasonal mean uptake coefficient of NH_3_ (γ_NH3_).** The box plots of γ_NH3_ in Shijiazhuang in different seasons.

**Supplementary Fig. 4**. **The sensitivity** **regime of NH_4_NO_3_ formation**. Particle phase fraction of (a) total nitrate, ɛ_NO3-_ and (b) total ammonium, ɛ_NH4+_, versus aerosol pH in Shijiazhuang. The equilibrium concentrations of NO_3_^-^, HNO_3_, NH_4_^+^ and NH_3_ in particle- and gas-phase were calculated using the ISORRPIA-II model.

**Supplementary Fig. 5**. **The variations of organic matter (OM) and SO_4_^2-^**. The ratio of OM/SO_4_^2-^ in Shijiazhuang and Beijing.

**Supplementary Fig. 6**. **The variation of nitrogen oxidation ratio (NOR).** The probability distribution and the probability-weighted NOR in Shijiazhuang.

**Supplementary Fig. 7. The variations of aerosol water content (AWC) and temperature (T).** The probability distribution of (a) AWC and (b) T in Shijiazhuang.

**Supplementary Fig. 8**. **Top 10 factors affect aerosol pH.** The relative importance of the top ten factors to aerosol pH simulated by random forest model.

**Supplementary Fig. 9.** **The fraction of the m/z 44 fragment in organic aerosol (OA)**. The nocturnal fraction of the m/z 44 in OA in Beijing.

**Supplementary Fig. 10.** **Identification of source number.** Variation of the Q_true_/Q_exp_ and d(Q_true_/Q_exp_)/dN as a function of the number of factors.

**Supplementary Fig. 11. The sources of PM_2.5_.** The source profiles of PM_2.5_ in Shijiazhuang. (a) coal combustion, (b) biomass burning, (c) traffic, (d) secondary nitrate, (e) dust, (f) secondary sulfate, and (g) industry.

**Supplementary Fig. 12. The normalized contributions of different PM_2.5_ sources.** The time series of different sources of PM_2.5_ in Shijiazhuang. (a) coal combustion, (b) biomass burning, (c) traffic, (d) secondary nitrate, (e) dust, (f) secondary sulfate, and (g) industry.

**Supplementary Fig. 13. The hourly and monthly mean source contribution of PM_2.5_.** (a) The diurnal and (b) monthly variations of different sources of PM_2.5_ in Shijiazhuang.

**Supplementary Fig. 14. The contribution variation of dust to PM_2.5_.** The relative contribution of dust emissions to PM_2.5_ mass concentration in Shijiazhuang. The equation of the dashed line is *y*=25.2-10.8*t* (*R*=-0.40).

**Supplementary Fig. 15. Influence of relative humidity (RH) on uptake coefficient of NH_3_ (γ_NH3_)**. The dependence of the probability-weighted γ_NH3_ on RH in Shijiazhuang.

**Supplementary Fi. 16. The contribution of daytime H_2_SO_4_ to uptake coefficient of NH_3_ (γ_NH3_)**. (a) The diurnal curve of estimated H_2_SO_4_ concentrations and (b) the box plots of γ_NH3_ considering the condensation of H_2_SO_4_ or not in Shijiazhuang.

**Supplementary Fig. 17. Influence of activity coefficient (γ_H+_) on aerosol pH**. (a) the dependence of activity coefficient on ionic strength and molarity of ions, (b) the correlation between the aerosol pH_γ_ and pH_F_, (c) and (d) the probability distribution of aerosol pH_γ_ and pH_F_ and the probability-weighted of aerosol pH_γ_ and pH_F_.


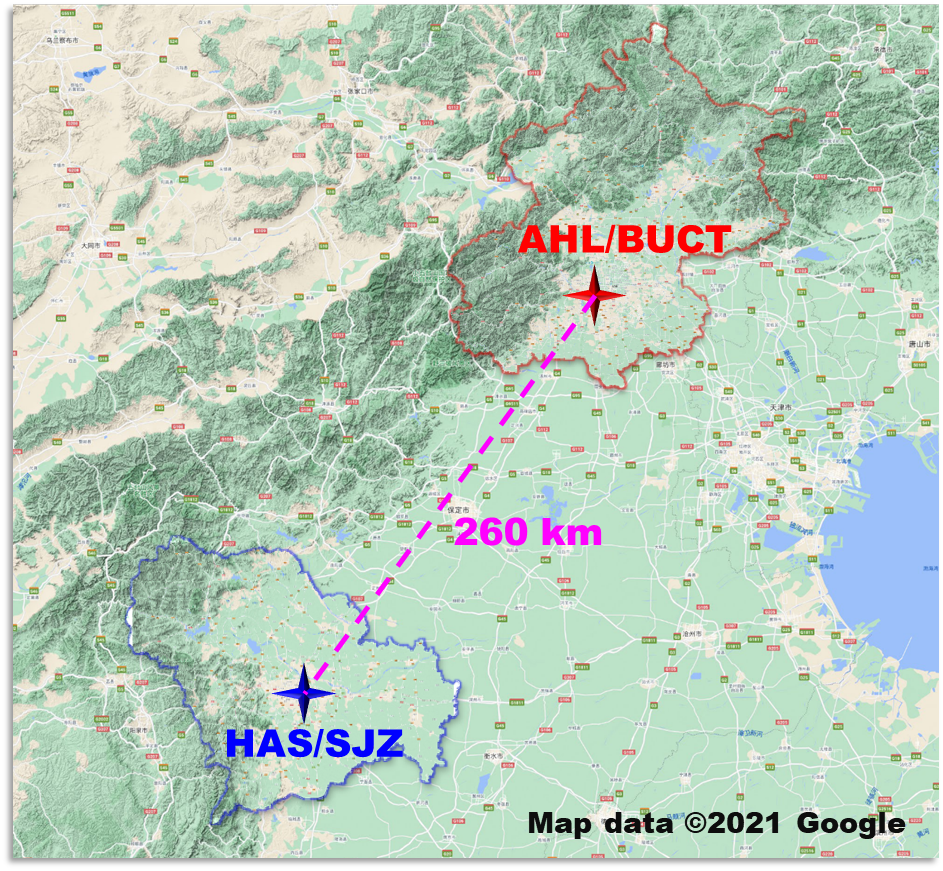


**Supplementary Fig. 18**. **The maps of the observation stations**. Map data ©2021 Google


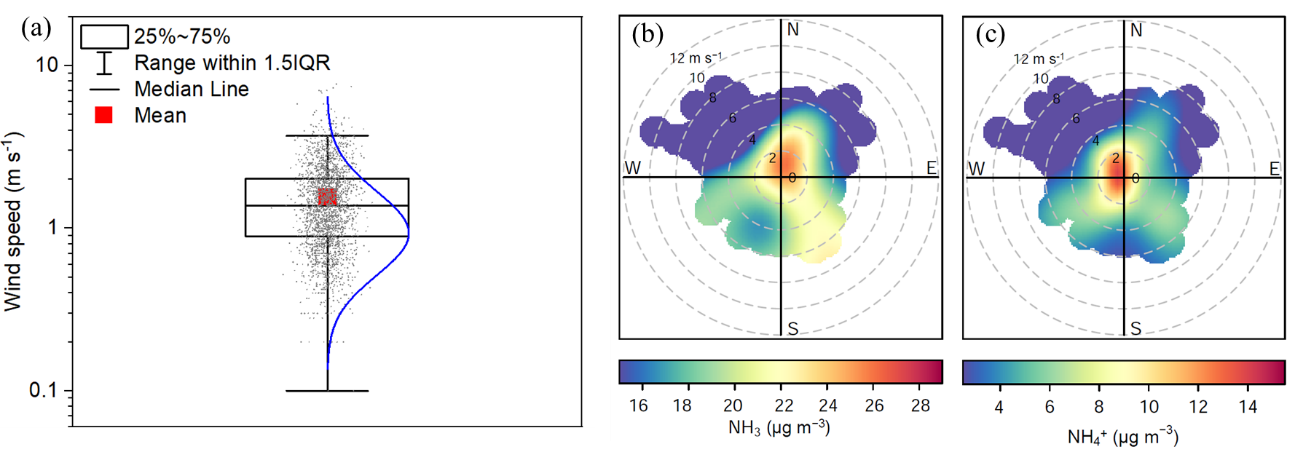


**Supplementary Fig. 19.** **Nocturnal wind speed and its influence on the concentrations of NH_3_ and NH_4_^+^.** (a) The distribution of nocturnal wind speed, and the distribution of nocturnal (b) NH_3_ and (c) NH_4_^+^ concentrations in the wind field in Shijiazhuang.

**Supplementary Fig. 20. The sensitivity of uptake coefficient of NH_3_ (γ_NH3_) on data-selection conditions**. The nocturnal γ_NH3_ (a) corresponding to temperature inversion and non-temperature inversion, (b) from 0:00 to 07:00 and from 18:00 to 23:00, and (c) constrained by a decrease and constant or increase in NH_3_ concentration in Shijiazhuang.

**Supplementary Fig. 21. Uncertainties of aerosol pH prediction.** (a) Loss during the training process and (b) prediction performance of random forest model for aerosol pH prediction.

**Supplementary references**

1. Huang R-J, Yang L, Cao J, Wang Q, Tie X, Ho K-F*, et al.* Concentration and sources of atmospheric nitrous acid (HONO) at an urban site in Western China. *Sci Total Environ* 2017, **593:** 165-172.

2. Su H, Cheng YF, Cheng P, Zhang YH, Dong S, Zeng LM*, et al.* Observation of nighttime nitrous acid (HONO) formation at a non-urban site during PRIDE-PRD2004 in China. *Atmos Environ* 2008, **42**(25)**:** 6219-6232.

3. Lammel G. Comment on “A DOAS study on the origin of nitrous acid at urban and non-urban sites”. *Atmos Environ* 1996, **30**(23)**:** 4101-4103.

4. Brown SS, Stark H, Ravishankara AR. Applicability of the steady state approximation to the interpretation of atmospheric observations of NO_3_ and N_2_O_5_. *J Geophys Res- Atmos* 2003, **108**(D17).

5. McDuffie EE, Fibiger DL, Dubé WP, Lopez-Hilfiker F, Lee BH, Thornton JA*, et al.* Heterogeneous N_2_O_5_ Uptake During Winter: Aircraft Measurements During the 2015 WINTER Campaign and Critical Evaluation of Current Parameterizations. *J Geophys Res- Atmos* 2018, **123**(8)**:** 4345-4372.

6. Bhattarai N, Wang S, Xu Q, Dong Z, Chang X, Jiang Y*, et al.* Sources of gaseous NH_3_ in urban Beijing from parallel sampling of NH3 and NH4+, their nitrogen isotope measurement and modeling. *Sci Total Environ* 2020, **747:** 141361.

7. Chang Y, Liu X, Deng C, Dore AJ, Zhuang G. Source apportionment of atmospheric ammonia before, during, and after the 2014 APEC summit in Beijing using stable nitrogen isotope signatures. *Atmos Chem Phys* 2016, **16**(18)**:** 11635-11647.

8. Dennis RL, Mathur R, Pleim JE, Walker JT. Fate of ammonia emissions at the local to regional scale as simulated by the Community Multiscale Air Quality model. *Atmos Pollut Res* 2010, **1**(4)**:** 207-214.

9. Pinder RW, Gilliland AB, Dennis RL. Environmental impact of atmospheric NH3 emissions under present and future conditions in the eastern United States. *Geophys Res Lett* 2008, **35**(12).
